# 3-Bromo-2-[4-(methyl­sulfan­yl)phen­yl]-5,6,7,8-tetra­hydro-1,3-benzo­thia­zolo[3,2-*a*]imidazole

**DOI:** 10.1107/S1600536814008976

**Published:** 2014-04-26

**Authors:** Alexander S. Bunev, Elena V. Sukhonosova, Vladimir E. Statsyuk, Gennady I. Ostapenko, Victor N. Khrustalev

**Affiliations:** a Department of Chemistry and Chemical Technology, Togliatti State University, 14 Belorusskaya St, Togliatti 445667, Russian Federation; b Department of Organic, Bioorganic and Medicinal Chemistry, Samara State University, 1 Akademician Pavlov St, Samara 443011, Russian Federation; c X-Ray Structural Centre, A.N. Nesmeyanov Institute of Organoelement Compounds, Russian Academy of Sciences, 28 Vavilov Street, B-334, Moscow 119991, Russian Federation

## Abstract

In the title mol­ecule, C_16_H_15_BrN_2_S_2_, the central imidazo[2,1-*b*]thia­zole fragment is almost planar (r.m.s. deviation = 0.012 Å), and the fused 5,6,7,8-tetra­hydro­benzene ring adopts an unsymmetrical half-chair conformation. The dihedral angle between the imidazo[2,1-*b*]thia­zole and benzene planes is 18.25 (4)°. The terminal methyl­sulfanyl substituent lies practically within the benzene plane [the dihedral angle between the corresponding planes is 7.20 (10)°] and is turned toward the C—Br bond. In the crystal, mol­ecules form infinite chains along [100] *via* secondary Br⋯N inter­actions [3.1861 (16) Å]. The chains are arranged at van der Waals distances.

## Related literature   

For applications of imidazo[2,1-*b*][1,3]benzo­thia­zoles, see: Ager *et al.* (1988 ▶); Sanfilippo *et al.* (1988 ▶); Barchéchath *et al.* (2005 ▶); Andreani *et al.* (2008 ▶); Chao *et al.* (2009 ▶); Kumbhare *et al.* (2011 ▶); Chandak *et al.* (2013 ▶). For the crystal structures of related compounds, see: Landreau *et al.* (2002 ▶); Adib *et al.* (2008 ▶); Fun, Asik *et al.* (2011 ▶); Fun, Hemamalini *et al.* (2011 ▶); Ghabbour *et al.* (2012 ▶); Bunev *et al.* (2013 ▶, 2014 ▶).
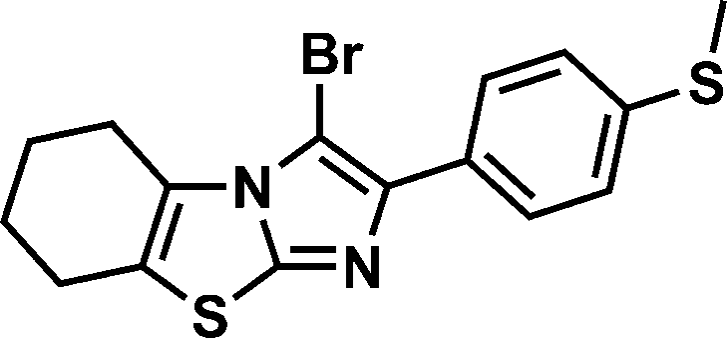


## Experimental   

### 

#### Crystal data  

C_16_H_15_BrN_2_S_2_
*M* *_r_* = 379.34
Triclinic, 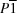

*a* = 7.3132 (3) Å
*b* = 7.5663 (3) Å
*c* = 14.4543 (7) Å
α = 95.033 (1)°
β = 97.188 (1)°
γ = 101.938 (1)°
*V* = 771.03 (6) Å^3^
*Z* = 2
Mo *K*α radiation
μ = 2.93 mm^−1^
*T* = 120 K
0.15 × 0.10 × 0.10 mm

#### Data collection  

Bruker APEXII CCD diffractometer
Absorption correction: multi-scan (*SADABS*; Bruker, 2003 ▶) *T*_min_ = 0.668, *T*_max_ = 0.758
10352 measured reflections
4508 independent reflections
3927 reflections with *I* > 2σ(*I*)
*R*_int_ = 0.025

#### Refinement  

*R*[*F* ^2^ > 2σ(*F* ^2^)] = 0.030
*wR*(*F* ^2^) = 0.073
*S* = 1.05
4508 reflections
191 parameters
H-atom parameters constrained
Δρ_max_ = 0.67 e Å^−3^
Δρ_min_ = −0.32 e Å^−3^

### 

Data collection: *APEX2* (Bruker, 2005 ▶); cell refinement: *SAINT* (Bruker, 2001 ▶); data reduction: *SAINT*; program(s) used to solve structure: *SHELXTL* (Sheldrick, 2008 ▶); program(s) used to refine structure: *SHELXTL*; molecular graphics: *SHELXTL*; software used to prepare material for publication: *SHELXTL*.

## Supplementary Material

Crystal structure: contains datablock(s) global, I. DOI: 10.1107/S1600536814008976/rk2426sup1.cif

Structure factors: contains datablock(s) I. DOI: 10.1107/S1600536814008976/rk2426Isup2.hkl

Supporting information file. DOI: 10.1107/S1600536814008976/rk2426Isup3.cml

CCDC reference: 998505

Additional supporting information:  crystallographic information; 3D view; checkCIF report

## Figures and Tables

**Fig. 1.**
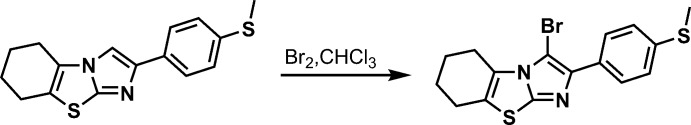
The synthesis of 3-bromo-2-[4-(methylsulfanyl)phenyl]-5,6,7,8-tetrahydro-1,3-benzothiazolo[3,2-a]imidazole.

**Fig. 2.**
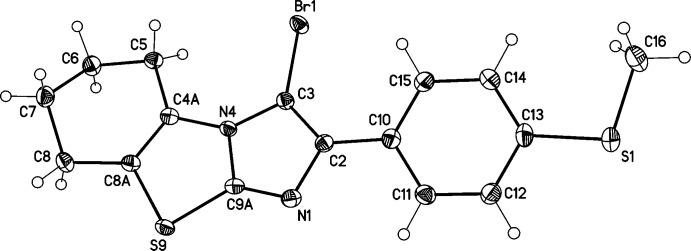
Molecular structure of I. Displacement ellipsoids are presented at the 50% probability level. H atoms are depicted as small spheres of arbitrary radius.

**Fig. 3.**
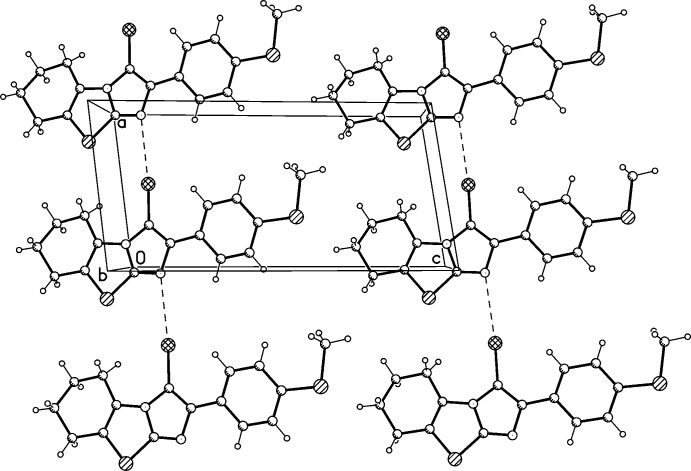
A portion of the crystal structure of I demonstrating the chains along [100]. The intermolecular secondary Br···N interactions are depicted by dashed lines.

## supplementary crystallographic information

### 1. Comment

Imidazo[2,1–*b*][1,3]benzothiazole are of great interest due to their biological properties. These compounds and their derivatives demonstrate the antitumor (Andreani *et al.*, 2008), antiallergic (Ager *et al.*, 1988), anesthetic (Sanfilippo *et al.*, 1988) and anti–cancer (Kumbhare *et al.*, 2011) activities as well as the inhibition activity of apoptosis in testiculargerm cells (Chandak *et al.*, 2013), lymphocytes (Barchéchath *et al.*, 2005), and FMS–like tyrosine kinase–3 (FLT3) (Chao *et al.*, 2009).

In this work, a new halogensubstituted 5,6,7,8–tetrahydrobenzo[d]imidazo[2,1–*b*]thiazole, C_16_H_15_BrN_2_S_2_, I, was prepared by the reaction of 5,6,7,8–tetrahydrobenzo[d]imidazo[2,1–*b*]thiazole with bromine at room temperature (Fig. 1), and its structure was unambiguously established by the X–ray diffraction study (Fig. 2).

In the title molecule (I) the central imidazo[2,1–*b*]thiazole fragment is almost planar (r.m.s. deviation = 0.012 Å), and the fused 5,6,7,8-tetrahydrobenzene ring adopts an unsymmetrical *half–chair* conformation (the C6 and C7 carbon atoms are out of the plane passed through the other atoms of the ring by -0.246 (2) and 0.508 (2) Å, respectively). The bond lengths and angles within the molecule of I are in a good agreement with those found in the related compounds (Landreau *et al.*, 2002; Adib *et al.*, 2008; Fun, Asik *et al.*, 2011; Fun, Hemamalini *et al.*, 2011; Ghabbour *et al.*, 2012; Bunev *et al.*, 2013, 2014). The dihedral angle between the imidazo[2,1–*b*]thiazole and benzene planes is 18.25 (4)°. The terminal methylthio substituent lies practically within the benzene plane (the dihedral angle between the corresponding planes is 7.20 (10)°) and is turned toward the C—Br bond.

In the crystal, the molecules of I form infinite chains along [100] by intermolecular secondary Br1···N1^i^ interactions (3.1861 (16) Å) (Fig. 3). The chains are arranged at van der Waals distances. Symmetry code: (i) 1 + *x*, *y*, *z*.

### 2. Experimental

A solution of bromine (139 µL, 430.4 mg, 2.69 mmol) in dry CHCl_3_ (10 mL) was added to a solutions 2–(4–(methylthio)phenyl)–5,6,7,8–tetrahydrobenzo[d]imidazo[2,1–b]thiazole (808.3 mg, 2.69 mmol) in dry CHCl_3_ (30 mL). The reaction mixture was stirred at room temperature for 3 h. the solvent was evaporated from the reaction mixture on rotavapor. The crude product was diluted with 5% solution Na_2_CO_3_ in water (25 mL). The precipitate was filtered and crystallized from *DMF*. Yield is 75%. The single–crystal of the product I was obtained by slow crystallization from *DMF*. M.p. = 439–441 K. IR (KBr), ν/cm^-1^: 3131, 3073, 1580, 1523, 1501, 1337, 1144, 815, 714. ^1^H NMR (600 MHz, *DMSO*–*d*_6_, 304 K): 7.63 (d, 2H, *J* = 8.9), 7.54 (d, 2H, *J* = 8.9), 3.39–3.33 (m, 2H), 3.02–2.96 (m, 2H), 2.45 (s, 3H), 1.91–1.81 (m, 4H). Anal. Calcd for C_16_H_15_BrN_2_S: C, 50.66; H, 3.99. Found: C, 50.57; H, 4.08.

### 3. Refinement

All hydrogen atoms were placed in the calculated positions with C—H = 0.95–0.99 Å and refined in the riding model with fixed isotropic displacement parameters: *U*_iso_(H) = 1.5*U*_eq_(C) for the methyl group and *U*_iso_(H) = 1.2*U*_eq_(C) for the other groups.

### Crystal data

**Table:**

C_16_H_15_BrN_2_S_2_	*Z* = 2
*M**_r_* = 379.34	*F*(000) = 384
Triclinic, *P*1	*D*_x_ = 1.634 Mg m^−^^3^
Hall symbol: -P 1	Mo *K*α radiation, λ = 0.71073 Å
*a* = 7.3132 (3) Å	Cell parameters from 4428 reflections
*b* = 7.5663 (3) Å	θ = 2.8–32.3°
*c* = 14.4543 (7) Å	µ = 2.93 mm^−^^1^
α = 95.033 (1)°	*T* = 120 K
β = 97.188 (1)°	Prism, colourless
γ = 101.938 (1)°	0.15 × 0.10 × 0.10 mm
*V* = 771.03 (6) Å^3^	

### Data collection

**Table:**

Bruker APEXII CCD diffractometer	4508 independent reflections
Radiation source: fine-focus sealed tube	3927 reflections with *I* > 2σ(*I*)
Graphite monochromator	*R*_int_ = 0.025
φ and ω scans	θ_max_ = 30.0°, θ_min_ = 2.8°
Absorption correction: multi-scan (*SADABS*; Bruker, 2003)	*h* = −10→10
*T*_min_ = 0.668, *T*_max_ = 0.758	*k* = −10→10
10352 measured reflections	*l* = −20→20

### Refinement

**Table:**

Refinement on *F*^2^	Primary atom site location: structure-invariant direct methods
Least-squares matrix: full	Secondary atom site location: difference Fourier map
*R*[*F*^2^ > 2σ(*F*^2^)] = 0.030	Hydrogen site location: inferred from neighbouring sites
*wR*(*F*^2^) = 0.073	H-atom parameters constrained
*S* = 1.05	*w* = 1/[σ^2^(*F*_o_^2^) + (0.0377*P*)^2^ + 0.1351*P*] where *P* = (*F*_o_^2^ + 2*F*_c_^2^)/3
4508 reflections	(Δ/σ)_max_ = 0.001
191 parameters	Δρ_max_ = 0.67 e Å^−^^3^
0 restraints	Δρ_min_ = −0.32 e Å^−^^3^

### Special details

**Table:**

Geometry. All s.u.'s (except the s.u. in the dihedral angle between two l.s. planes) are estimated using the full covariance matrix. The cell s.u.'s are taken into account individually in the estimation of s.u.'s in distances, angles and torsion angles; correlations between s.u.'s in cell parameters are only used when they are defined by crystal symmetry. An approximate (isotropic) treatment of cell s.u.'s is used for estimating s.u.'s involving l.s. planes.
Refinement. Refinement of *F*^2^ against ALL reflections. The weighted *R*–factor w*R* and goodness of fit *S* are based on *F*^2^, conventional *R*–factors *R* are based on *F* with *F* set to zero for negative *F*^2^. The threshold expression of *F*^2^ > 2σ(*F*^2^) is used only for calculating *R*–factors(gt) etc. and is not relevant to the choice of reflections for refinement. *R*–factors based on *F*^2^ are statistically about twice as large as those based on *F*, and *R*–factors based on ALL data will be even larger.

### Fractional atomic coordinates and isotropic or equivalent isotropic displacement parameters (Å^2^)

**Table:**

	*x*	*y*	*z*	*U*_iso_*/*U*_eq_	
Br1	0.52846 (2)	0.33371 (2)	0.103656 (13)	0.01782 (6)	
S1	0.31673 (8)	0.75383 (8)	0.55165 (4)	0.02712 (12)	
N1	−0.0318 (2)	0.3490 (2)	0.10823 (11)	0.0169 (3)	
C2	0.1565 (3)	0.3866 (2)	0.15151 (13)	0.0150 (3)	
C3	0.2697 (2)	0.3284 (2)	0.09002 (13)	0.0146 (3)	
N4	0.1494 (2)	0.2541 (2)	0.00724 (10)	0.0136 (3)	
C4A	0.1551 (3)	0.1754 (2)	−0.08424 (13)	0.0147 (3)	
C5	0.3302 (3)	0.1399 (2)	−0.11797 (13)	0.0159 (3)	
H5A	0.3921	0.0696	−0.0739	0.019*	
H5B	0.4195	0.2568	−0.1199	0.019*	
C6	0.2819 (3)	0.0326 (3)	−0.21646 (14)	0.0194 (4)	
H6A	0.3978	0.0462	−0.2464	0.023*	
H6B	0.2372	−0.0981	−0.2106	0.023*	
C7	0.1308 (3)	0.0964 (3)	−0.27918 (13)	0.0208 (4)	
H7A	0.1110	0.0295	−0.3429	0.025*	
H7B	0.1735	0.2276	−0.2844	0.025*	
C8	−0.0560 (3)	0.0635 (3)	−0.23845 (13)	0.0186 (4)	
H8A	−0.1458	0.1253	−0.2726	0.022*	
H8B	−0.1133	−0.0684	−0.2457	0.022*	
C8A	−0.0175 (3)	0.1366 (2)	−0.13656 (13)	0.0154 (3)	
S9	−0.19453 (6)	0.18940 (6)	−0.07481 (3)	0.01700 (10)	
C9A	−0.0272 (2)	0.2722 (2)	0.02379 (13)	0.0153 (3)	
C10	0.2059 (3)	0.4782 (2)	0.24789 (13)	0.0164 (3)	
C11	0.0607 (3)	0.4836 (3)	0.30255 (14)	0.0191 (4)	
H11	−0.0658	0.4267	0.2767	0.023*	
C12	0.0991 (3)	0.5705 (3)	0.39362 (14)	0.0204 (4)	
H12	−0.0014	0.5733	0.4291	0.024*	
C13	0.2850 (3)	0.6542 (3)	0.43388 (13)	0.0199 (4)	
C14	0.4292 (3)	0.6527 (3)	0.37994 (14)	0.0232 (4)	
H14	0.5554	0.7108	0.4058	0.028*	
C15	0.3898 (3)	0.5664 (3)	0.28802 (14)	0.0217 (4)	
H15	0.4900	0.5676	0.2520	0.026*	
C16	0.5684 (3)	0.8198 (3)	0.58283 (16)	0.0302 (5)	
H16A	0.6001	0.8615	0.6502	0.045*	
H16B	0.6187	0.9185	0.5472	0.045*	
H16C	0.6242	0.7154	0.5682	0.045*	

### Atomic displacement parameters (Å^2^)

**Table:**

	*U*^11^	*U*^22^	*U*^33^	*U*^12^	*U*^13^	*U*^23^
Br1	0.01313 (9)	0.02030 (10)	0.01932 (10)	0.00599 (7)	−0.00109 (6)	−0.00219 (7)
S1	0.0271 (3)	0.0327 (3)	0.0173 (2)	0.0007 (2)	0.0029 (2)	−0.0057 (2)
N1	0.0157 (7)	0.0163 (7)	0.0187 (8)	0.0026 (6)	0.0042 (6)	0.0015 (6)
C2	0.0154 (8)	0.0140 (8)	0.0158 (8)	0.0029 (7)	0.0033 (7)	0.0023 (6)
C3	0.0134 (8)	0.0155 (8)	0.0154 (8)	0.0051 (7)	0.0010 (6)	0.0008 (6)
N4	0.0109 (7)	0.0153 (7)	0.0145 (7)	0.0034 (6)	0.0011 (5)	0.0010 (6)
C4A	0.0145 (8)	0.0121 (8)	0.0175 (9)	0.0030 (6)	0.0023 (7)	0.0016 (6)
C5	0.0140 (8)	0.0160 (8)	0.0186 (9)	0.0050 (7)	0.0036 (7)	0.0004 (7)
C6	0.0195 (9)	0.0217 (9)	0.0179 (9)	0.0083 (8)	0.0030 (7)	−0.0010 (7)
C7	0.0221 (9)	0.0247 (10)	0.0159 (9)	0.0070 (8)	0.0025 (7)	−0.0004 (7)
C8	0.0178 (9)	0.0193 (9)	0.0167 (9)	0.0036 (7)	−0.0016 (7)	−0.0013 (7)
C8A	0.0136 (8)	0.0140 (8)	0.0182 (8)	0.0025 (7)	0.0028 (7)	0.0007 (6)
S9	0.01080 (19)	0.0210 (2)	0.0186 (2)	0.00355 (17)	0.00092 (16)	0.00057 (17)
C9A	0.0119 (8)	0.0160 (8)	0.0186 (8)	0.0030 (7)	0.0033 (7)	0.0031 (7)
C10	0.0192 (9)	0.0129 (8)	0.0167 (8)	0.0027 (7)	0.0028 (7)	0.0010 (6)
C11	0.0176 (9)	0.0174 (9)	0.0208 (9)	0.0017 (7)	0.0021 (7)	0.0001 (7)
C12	0.0211 (9)	0.0208 (9)	0.0193 (9)	0.0037 (8)	0.0051 (7)	0.0010 (7)
C13	0.0252 (10)	0.0187 (9)	0.0145 (8)	0.0037 (8)	0.0019 (7)	−0.0011 (7)
C14	0.0196 (9)	0.0252 (10)	0.0208 (10)	−0.0006 (8)	0.0014 (7)	−0.0028 (8)
C15	0.0205 (9)	0.0235 (10)	0.0194 (9)	0.0009 (8)	0.0063 (7)	−0.0012 (8)
C16	0.0286 (11)	0.0311 (12)	0.0249 (11)	−0.0003 (9)	−0.0030 (9)	−0.0030 (9)

### Geometric parameters (Å, º)

**Table:**

Br1—C3	1.8693 (18)	C7—H7B	0.9900
S1—C13	1.7673 (19)	C8—C8A	1.498 (3)
S1—C16	1.793 (2)	C8—H8A	0.9900
N1—C9A	1.312 (2)	C8—H8B	0.9900
N1—C2	1.400 (2)	C8A—S9	1.7529 (19)
C2—C3	1.391 (2)	S9—C9A	1.7354 (19)
C2—C10	1.467 (3)	C10—C15	1.399 (3)
C3—N4	1.391 (2)	C10—C11	1.405 (3)
N4—C9A	1.374 (2)	C11—C12	1.387 (3)
N4—C4A	1.411 (2)	C11—H11	0.9500
C4A—C8A	1.349 (2)	C12—C13	1.403 (3)
C4A—C5	1.492 (2)	C12—H12	0.9500
C5—C6	1.537 (3)	C13—C14	1.389 (3)
C5—H5A	0.9900	C14—C15	1.396 (3)
C5—H5B	0.9900	C14—H14	0.9500
C6—C7	1.524 (3)	C15—H15	0.9500
C6—H6A	0.9900	C16—H16A	0.9800
C6—H6B	0.9900	C16—H16B	0.9800
C7—C8	1.537 (3)	C16—H16C	0.9800
C7—H7A	0.9900		
			
C13—S1—C16	103.71 (10)	C8A—C8—H8B	109.9
C9A—N1—C2	104.13 (15)	C7—C8—H8B	109.9
C3—C2—N1	110.13 (16)	H8A—C8—H8B	108.3
C3—C2—C10	130.54 (17)	C4A—C8A—C8	124.20 (17)
N1—C2—C10	119.33 (16)	C4A—C8A—S9	113.31 (14)
C2—C3—N4	106.03 (15)	C8—C8A—S9	122.44 (13)
C2—C3—Br1	131.99 (14)	C9A—S9—C8A	89.99 (9)
N4—C3—Br1	121.98 (13)	N1—C9A—N4	114.32 (16)
C9A—N4—C3	105.38 (15)	N1—C9A—S9	134.58 (14)
C9A—N4—C4A	114.32 (15)	N4—C9A—S9	111.08 (13)
C3—N4—C4A	140.26 (16)	C15—C10—C11	117.62 (17)
C8A—C4A—N4	111.27 (16)	C15—C10—C2	123.61 (17)
C8A—C4A—C5	124.77 (17)	C11—C10—C2	118.73 (17)
N4—C4A—C5	123.97 (16)	C12—C11—C10	121.15 (18)
C4A—C5—C6	110.25 (15)	C12—C11—H11	119.4
C4A—C5—H5A	109.6	C10—C11—H11	119.4
C6—C5—H5A	109.6	C11—C12—C13	120.61 (18)
C4A—C5—H5B	109.6	C11—C12—H12	119.7
C6—C5—H5B	109.6	C13—C12—H12	119.7
H5A—C5—H5B	108.1	C14—C13—C12	118.75 (18)
C7—C6—C5	112.55 (15)	C14—C13—S1	124.86 (16)
C7—C6—H6A	109.1	C12—C13—S1	116.38 (15)
C5—C6—H6A	109.1	C13—C14—C15	120.45 (19)
C7—C6—H6B	109.1	C13—C14—H14	119.8
C5—C6—H6B	109.1	C15—C14—H14	119.8
H6A—C6—H6B	107.8	C14—C15—C10	121.38 (18)
C6—C7—C8	110.44 (16)	C14—C15—H15	119.3
C6—C7—H7A	109.6	C10—C15—H15	119.3
C8—C7—H7A	109.6	S1—C16—H16A	109.5
C6—C7—H7B	109.6	S1—C16—H16B	109.5
C8—C7—H7B	109.6	H16A—C16—H16B	109.5
H7A—C7—H7B	108.1	S1—C16—H16C	109.5
C8A—C8—C7	109.02 (15)	H16A—C16—H16C	109.5
C8A—C8—H8A	109.9	H16B—C16—H16C	109.5
C7—C8—H8A	109.9		
			
C9A—N1—C2—C3	−0.4 (2)	C8—C8A—S9—C9A	−175.86 (16)
C9A—N1—C2—C10	178.60 (16)	C2—N1—C9A—N4	0.5 (2)
N1—C2—C3—N4	0.2 (2)	C2—N1—C9A—S9	−178.31 (16)
C10—C2—C3—N4	−178.66 (17)	C3—N4—C9A—N1	−0.4 (2)
N1—C2—C3—Br1	−178.84 (14)	C4A—N4—C9A—N1	−178.63 (15)
C10—C2—C3—Br1	2.3 (3)	C3—N4—C9A—S9	178.71 (12)
C2—C3—N4—C9A	0.07 (19)	C4A—N4—C9A—S9	0.45 (19)
Br1—C3—N4—C9A	179.24 (12)	C8A—S9—C9A—N1	177.7 (2)
C2—C3—N4—C4A	177.6 (2)	C8A—S9—C9A—N4	−1.11 (14)
Br1—C3—N4—C4A	−3.2 (3)	C3—C2—C10—C15	17.2 (3)
C9A—N4—C4A—C8A	0.7 (2)	N1—C2—C10—C15	−161.53 (18)
C3—N4—C4A—C8A	−176.6 (2)	C3—C2—C10—C11	−164.91 (19)
C9A—N4—C4A—C5	−179.12 (16)	N1—C2—C10—C11	16.3 (3)
C3—N4—C4A—C5	3.5 (3)	C15—C10—C11—C12	−1.1 (3)
C8A—C4A—C5—C6	−6.7 (3)	C2—C10—C11—C12	−179.12 (17)
N4—C4A—C5—C6	173.18 (16)	C10—C11—C12—C13	−0.5 (3)
C4A—C5—C6—C7	39.6 (2)	C11—C12—C13—C14	1.7 (3)
C5—C6—C7—C8	−63.1 (2)	C11—C12—C13—S1	−177.96 (15)
C6—C7—C8—C8A	49.1 (2)	C16—S1—C13—C14	−6.6 (2)
N4—C4A—C8A—C8	175.79 (16)	C16—S1—C13—C12	173.04 (16)
C5—C4A—C8A—C8	−4.3 (3)	C12—C13—C14—C15	−1.2 (3)
N4—C4A—C8A—S9	−1.6 (2)	S1—C13—C14—C15	178.47 (16)
C5—C4A—C8A—S9	178.26 (14)	C13—C14—C15—C10	−0.5 (3)
C7—C8—C8A—C4A	−17.4 (3)	C11—C10—C15—C14	1.7 (3)
C7—C8—C8A—S9	159.79 (14)	C2—C10—C15—C14	179.55 (18)
C4A—C8A—S9—C9A	1.59 (15)		
